# Screening of Drugs to Treat 8p11 Myeloproliferative Syndrome Using Patient-Derived Induced Pluripotent Stem Cells with Fusion Gene CEP110-FGFR1

**DOI:** 10.1371/journal.pone.0120841

**Published:** 2015-03-24

**Authors:** Shohei Yamamoto, Makoto Otsu, Emiko Matsuzaka, Chieko Konishi, Haruna Takagi, Sachiyo Hanada, Shinji Mochizuki, Hiromitsu Nakauchi, Kohzoh Imai, Kohichiro Tsuji, Yasuhiro Ebihara

**Affiliations:** 1 Department of Pediatric Hematology/Oncology, Center for Stem Cell Biology and Regenerative Medicine, Institute of Medical Science, The University of Tokyo, Tokyo, Japan; 2 Division of Stem Cell Processing, Center for Stem Cell Biology and Regenerative Medicine, Institute of Medical Science, The University of Tokyo, Tokyo, Japan; 3 Stem Cell Bank, Center for Stem Cell Biology and Regenerative Medicine, Institute of Medical Science, The University of Tokyo, Tokyo, Japan; 4 Division of Stem Cell Therapy, Center for Stem Cell Biology and Regenerative Medicine, Institute of Medical Science, The University of Tokyo, Tokyo, Japan; 5 Center for Antibody and Vaccine, IMSUT Hospital, Center for Stem Cell Biology and Regenerative Medicine, Institute of Medical Science, The University of Tokyo, Tokyo, Japan; 6 National Hospital Organization Shinshu Ueda Medical Center, Ueda, Japan; 7 Division of Advanced Regenerative Medicine, Center for Stem Cell Biology and Regenerative Medicine, Institute of Medical Science, The University of Tokyo, Tokyo, Japan; Josep Carreras Leukaemia Research Institute, University of Barcelona, SPAIN

## Abstract

Induced pluripotent stem (iPS) cells provide powerful tools for studying disease mechanisms and developing therapies for diseases. The 8p11 myeloproliferative syndrome (EMS) is an aggressive chronic myeloproliferative disorder (MPD) that is caused by constitutive activation of *fibroblast growth factor receptor 1*. EMS is rare and, consequently, effective treatment for this disease has not been established. Here, iPS cells were generated from an EMS patient (EMS-iPS cells) to assist the development of effective therapies for EMS. When iPS cells were co-cultured with murine embryonic stromal cells, EMS-iPS cells produced more hematopoietic progenitor and hematopoietic cells, and CD34^+^ cells derived from EMS-iPS cells exhibited 3.2–7.2-fold more macrophage and erythroid colony forming units (CFUs) than those derived from control iPS cells. These data indicate that EMS-iPS cells have an increased hematopoietic differentiation capacity, which is characteristic of MPDs. To determine whether a tyrosine kinase inhibitor (TKI) could suppress the increased number of CFUs formed by EMS-iPS-induced CD34^+^ cells, cells were treated with one of four TKIs (CHIR258, PKC 412, ponatinib, and imatinib). CHIR258, PKC 412, and ponatinib reduced the number of CFUs formed by EMS-iPS-induced CD34^+^ cells in a dose-dependent manner, whereas imatinib did not. Similar effects were observed on primary peripheral blood cells (more than 90% of which were blasts) isolated from the patient. This study provides evidence that the EMS-iPS cell line is a useful tool for the screening of drugs to treat EMS and to investigate the mechanism underlying this disease.

## Introduction

The 8p11 myeloproliferative syndrome (EMS), also known as stem cell leukemia/lymphoma, is an aggressive chronic myeloproliferative disorder (MPD) that is frequently accompanied by T- or B-lymphoblastic lymphoma and rapidly transforms into acute myeloid leukemia (AML) [1] [2] [3]. The hallmark of this disease is a reciprocal translocation involving the *fibroblast growth factor receptor 1* (*FGFR1*) gene, which is located on chromosome 8p11, encodes a tyrosine kinase, and has various gene partners [3]. The most common gene partner of *FGFR1* is *ZNF198*, which is located on chromosome 13q12 [2]. The second most common gene partner of *FGFR1* is *CEP110*, which is located on chromosome 9p33, and fewer than 20 cases of this translocation have been reported [3] [4]. Constitutive activation of *FGFR1* underlies the pathogenesis of EMS [5].

The incidence of EMS is low, which has limited study of the mechanism underlying this disease as well as drug screening to identify treatments. Recently generation of pluripotent stem cell from blood cells became feasible [6–8]. We thus established an induced pluripotent stem (iPS) cell line from a patient with EMS who had the t(8;9)(p12;q33) translocation and expressed a FGFR1/CEP110 fusion transcript. This cell line was termed ‘EMS-iPS’. EMS-iPS cells had an enhanced hematopoietic differentiation capacity favoring myeloid differentiation, recapitulating the cellular phenotype of MPDs. Three tyrosine kinase inhibitors (TKIs) reduced the number of colony forming units (CFUs) formed by EMS-iPS-induced CD34^+^ cells in a dose-dependent manner. The EMS-iPS cell line provides a powerful tool for studying the cellular and molecular mechanisms underlying EMS and developing treatments for this disease.

## Materials and Methods

Human samples were used in accordance with the Declaration of Helsinki. The study was approved by the ethics committee of The Institute of Medical Science, The University of Tokyo (protocol #25-3-0701). Written informed consent for samples to be used for research purposes was obtained from the patient’s parents. Animal experiments and the use of viral vectors were approved by the ethics committees of The Institute of Medical Science and the School of Medicine at The University of Tokyo.

### Case

A 17-year-old male was admitted to hospital owing to lymphadenopathy and leukocytosis. He was first diagnosed with AML (FAB M0) with the t(8;9)(p12;q33) translocation, and received idarubicin and cytarabine as an induction therapy. He was referred to our hospital for further treatment. Multiple lymphadenopathy and hepatosplenomegaly were identified on his admission to our hospital. In a bone marrow (BM) aspirate, 56.4% of cells were myeloblasts, which were positive for CD7, CD13, CD33, CD34, and HLA-DR. Karyotype analysis revealed the t(8;9)(p12;q33) translocation, and reverse transcription (RT)-PCR analysis detected a chimeric FGFR1/CEP110 fusion transcript. A lymphoid node biopsy specimen showed diffuse infiltration of small lymphoblasts, which were positive for cytoplasmic CD3, CD5, CD7, and terminal deoxynucleotidyl transferase. Karyotype and RT-PCR analyses of the lymphoid node biopsy specimen revealed the same abnormalities as detected in the BM aspirate. Based on these results, the patient was diagnosed with EMS. He had never achieved complete remission even though he had received several courses of chemotherapy. Some of his leukemic blasts exhibited other abnormalities, including trisomy 21 (S1 Table). Therefore, allogeneic BM transplantation was performed 6 months after the patient was diagnosed with EMS. He achieved complete chimerism on day 31 with grade III acute graft-versus-host disease; however, the FGFR1/CEP110 fusion transcript was still detected. He developed hematological relapse on day 68 and died on day 92. This case was previously reported [9].

### Chemicals

CHIR258 (TKI-258/Dovitinib) and ponatinib (AP24534) were purchased from Selleck Chemicals, PKC 412 was purchased from R&D Systems, and imatinib was purchased from LC Laboratories. All inhibitors were dissolved in dimethyl sulfoxide to a concentration of 10 mM and were stored at −20°C in single-use aliquots. CHIR258, PKC 412, and ponatinib reportedly have the potential to treat EMS [10] [11] [12] [13] [10–14].

### Generation and culture of EMS-iPS cells

BM mononuclear cells (MNCs) from the EMS patient after 5^th^ course of chemotherapy were separated using Ficoll-Hypaque density gradient centrifugation and were maintained in Eagle’s minimum essential medium (MEM) containing 10% fetal bovine serum (FBS) (Hyclone). EMS-iPS cells (SPH-0809 line) were established from these BM MNCs using retroviruses harboring four reprogramming factors (OCT4, SOX2, KLF4, and c-MYC). pMX retroviral vectors were provided by Dr. T. Kitamura (The University of Tokyo, Tokyo, Japan). Retroviral supernatants to establish EMS-iPS cells were obtained using a 293 GPG system (provided by Dr. R.C. Mulligan, Boston Children’s Hospital, Boston, MA) [15]. Established EMS-iPS cells were maintained as described previously [16]. EMS-iPS cells were passaged every 5–7 days on mitomycin C-treated MEF feeder cells in EMS-iPS cell maintenance medium, which consisted of a 1:1 ratio of Dulbecco’s MEM and Ham’s nutrient mixture F-12 (Sigma) supplemented with 0.1 mM 2-mercaptoethanol (2-ME; Wako), 2 mM L-glutamine (Wako), 1% non-essential amino acid solution (Invitrogen), 4 ng/ml human basic fibroblast growth factor (Wako), and 20% knockout serum replacement (Invitrogen) [17]. Control iPS cell clones, control 1 (201B7) and control 2 (TkDN4-M), were gifts from Drs. Yamanaka and Eto (Kyoto University, Kyoto), respectively [16] [18].

### Bisulfite Sequencing

Genomic DNA was treated with a MethylEasy Xceed Rapid DNA Bisulphite Modification kit (Human Genetic Signatures) according to the manufacturer’s recommendations. The promoter regions of OCT3/4 and NANOG were amplified by PCR using EpiTaq HS (Takara). The PCR products were cloned into pGEM-T-Easy vector (Promega) and sequenced. The primer sequences used for the PCR are shown in S2 Table. Data was analyzed and presented by QUMA.

### Immunohistochemistry

Immunochemical staining of iPS cell colonies was performed as described previously [19]. Briefly, cells were fixed with phosphate-buffered solution (PBS) containing 4% paraformaldehyde at 4°C overnight, and then permeabilized and blocked in PBS containing 5% skimmed milk, 0.1% Triton X-100, and 5% normal goat serum (Millipore) for 15 min at room temperature. Goat anti-human Nanog and OCT3/4 polyclonal primary antibodies (R&D) and mouse anti-human SOX2 and PODXL monoclonal primary antibodies (R&D) were used. Fluorescein isothiocyanate- and carbocyanine 3-conjugated secondary antibodies (Jackson ImmunoResearch) were used.

### PCR of genomic DNA

Genomic DNA was extracted from cells originated patient’s bone marrow and iPS cells using QIAamp DNA Mini Kit (QIAGEN). The CEPF2-FGFR_ER primer pair used to amplify the CEP110-FGFR1 genomic fusion locus is described in S2 Table. To determine the fusion point, the PCR products ware cloned into pCR4Blunt-TOPO (Life Technologies as described by the manufacturer) and sequenced (S1 Fig.).

### RT-PCR

Total RNA was extracted from cells using an illustra RNAspin Mini RNA Isolation Kit (GE Healthcare), and complementary DNA (cDNA) was synthesized using an oligo(dT) primer and an iScript select cDNA Synthesis Kit (Bio-Rad). PCR was performed using LA Taq (TAKARA, Japan) to determine the expression levels of *SOX2*, *PODXL*, *C-myc*, *Nanog*, and *OCT3/4*. *GAPDH* was used as the reference gene. The primer sequences are listed in S2 Table.

### Teratoma formation

To determine the pluripotential of iPS cells, teratomas formation assay was performed following previous report [20]. Briefly inoculating 1 × 10^6^ cells were injected into the testes of mice with severe combined immunodeficiency (Charles River). After 9–13 weeks, resected teratomas were fixed in 20% formalin, processed for paraffin sectioning, and stained with hematoxylin and eosin.

### Co-culture of iPS cells with AGM-S3 cells

iPS cells were maintained and passaged weekly on mitomycin C-treated MEF feeder cells, as described previously [17] [19] [21]. To evaluate the potential of iPS cells to differentiate into hematopoietic cells, iPS cells were co-cultured with the murine stromal cell line, AGM-S3 [17] [22] [23]. Briefly, 50 undifferentiated iPS cell colonies (each consisting of approximately 1 × 10^3^ cells) were transferred to each well of a 6-well plate (Sumitomo Bakelite Co), which contained confluent 15 Gy-irradiated AGM-S3 cells (approximately 2–3 × 10^5^ cells per well). Cells were co-cultured in iPS cell maintenance medium for 3 days. This culture medium was then replaced with hematopoietic differentiation medium (Iscove’s Modified Dulbecco’s Medium; IMDM, containing 10% FBS, 3 mM L-glutamine, 5.5 mg/ml human transferrin, 50 ng/ml ascorbic acid, 0.1 mM 2-ME, 0.1 mM non-essential amino acids, and 100 ng/ml human vascular endothelial growth factor). Thereafter, the medium was changed daily. After 12 days, proliferating cells and those with a cobblestone morphology were observed. Cells were harvested, and CD34^+^ cells were isolated using the Dynal CD34 Progenitor Cell Selection System (Invitrogen).

### Hematopoietic colony assay and suspension culture

A hematopoietic colony assay was performed in an aliquot of culture mixture, which contained 1.2% methylcellulose (Shin-Etsu Chemical, Japan), 30% FBS, 1% deionized fraction V bovine serum albumin (BSA), 0.1 mM 2-ME, α-Minimum Essential Medium (MEM), and a cytokine cocktail consisting of 100 ng/mL human stem cell factor (hSCF; Wako), 10 ng/mL human interleukin-3 (hIL-3; gifted by Kirin Brewery, Japan), 10 ng/mL human thrombopoietin (hTPO; gifted by Kirin Brewery), 10 ng/mL human granulocyte colony stimulating factor (G-CSF; gifted by Chugai Pharmaceutical Co., Japan), and 5 U/mL human erythropoietin (hEPO; gifted by Kirin Brewery), and were plated in a 35-mm suspension culture dish (BD falcon) and incubated at 37°C in a humidified atmosphere flushed with 5% CO_2_ in air. Colony types were determined according to established criteria on day 14 of culture [24].

In suspension culture, harvested CD34^+^ cells (1 × 10^4^ cells) were cocultured with irradiated confluent AGM-S3 cells medium containing IMDM, 10% FBS, 100 ng/mL hSCF, 10 ng/mL hIL-3, 10 ng/mL hTPO, and 10 ng/mL hG-CSF, 100 ng/ml hIL-6 (Wako), 5 U/mL EPO, 10ng/ml Fms-related tyrosine kinase 3 ligand (FL; Wako). The medium was replaced with an equivalent volume of fresh medium every 4 days. Living, non-adherent cells were counted following 0.4% trypan blue staining.

In some experiments, a TKI (CHIR258, PKC 412, ponatinib, or imatinib) was added to the culture medium, and the effects on colony formation in the hematopoietic colony assay or on the number of living cells in suspension cultures were examined.

### Flow cytometric analysis

Cells cocultured with AGM-S3 cells were dissociated with 0.05% trypsin-EDTA solution (Sigma-Aldrich) and filtered through a nylon screen to obtain a single-cell suspension. Flow cytometric analysis was performed using a FACSCalibur (BD Biosciences) and the data were analyzed using FlowJo Version 9.7.6 software (TreeStar). The following antibodies were used for the flow cytometric analysis: CD34 (clone 581; phycoerythrin (PE), BD), CD45 (clone ALB12; fluorescein isothiocyanate (FITC), Immunotech).

### Suspension culture of patient-derived primary cells

MNCs from peripheral blood (PB) (including > 90% blasts) of the EMS patient were cultured in conditioned medium from iPS cells cultured in the absence or presence of a TKI for 7 days. The number of living non-adherent cells was counted following 0.4% trypan blue staining.

### Microscopy

Images of cells were acquired using a microscope (IX70, Olympus) equipped with a camera module (DP70, Olympus), the appropriate objective, and DP Controller Software (Olympus).

### Statistical analysis

All data are presented as the mean ± standard deviation (SD). *P*-values of less than 0.05 were considered significant. Statistical analyses were performed using Prism software (GraphPad).

## Results

### Establishment of iPS cells from an EMS patient with the t(8;9)(p12;q33) translocation with fusion gene CEP110-FGFR1

**Fig 1:**
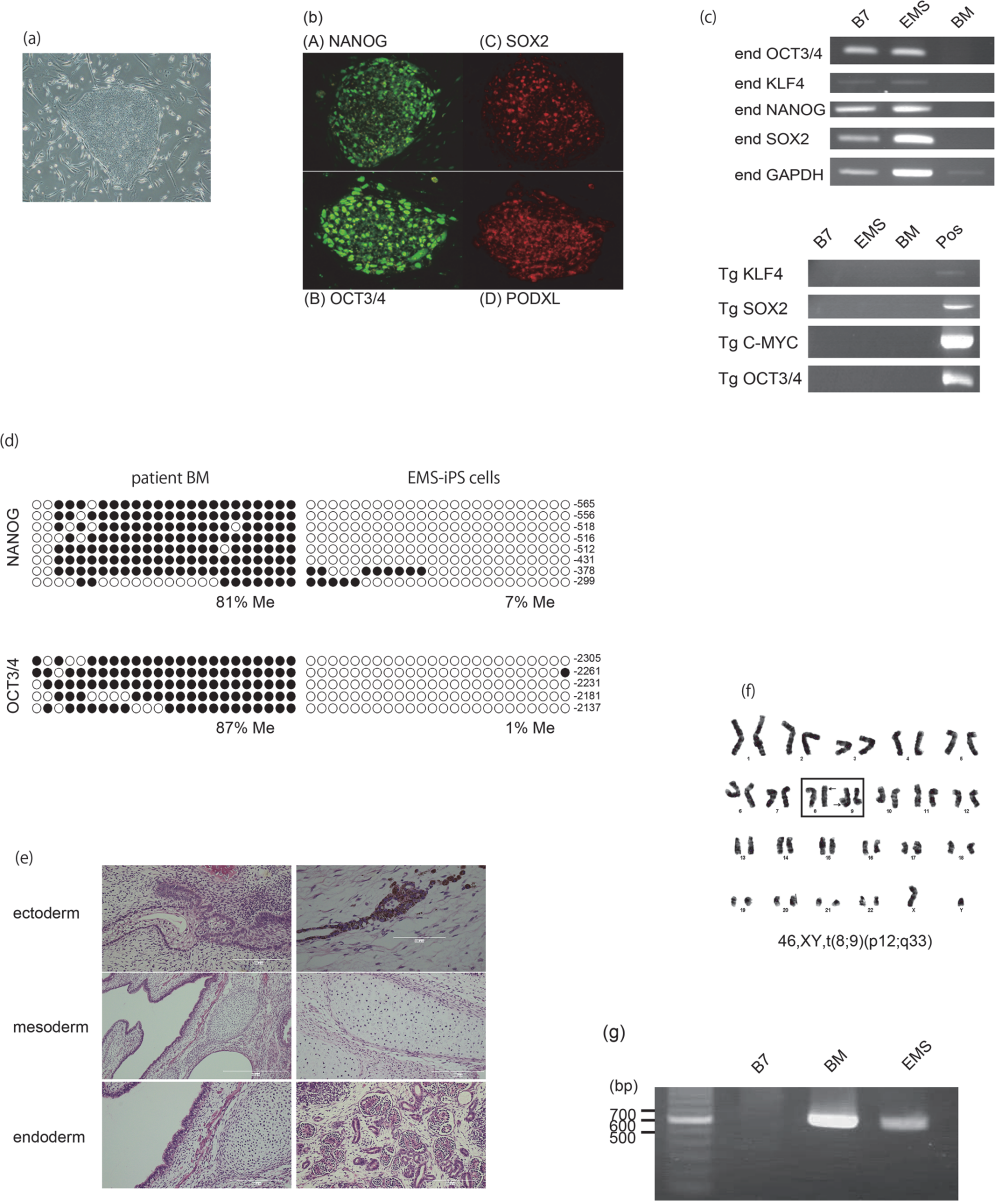
Generation of iPS cells from an EMS patient with the t(8;9)(p12;q33) translocation. (**a**) Morphology of EMS-iPS cells (×40 magnification). (**b**) Expression of the pluripotency markers Nanog (A), SOX2 (B), OCT3/4 (C), and PODXL (D) in EMS-iPS cells (×10 magnification). (**c**) Semi-quantitative RT-PCR analysis of the expression levels of endogenous (end) and viral-derived (Tg) reprogramming factors. Primary BM cells from the patient and control 201B7 human iPS cells (B7) are included as controls. (**d**) Bisulfite sequencing analyses of the *OCT3/4* and *NANOG* promoter regions in patient BM cells and EMS-iPS cells. White and black circles represent unmethylated and methylated (Me) CpG dinucleotides, respectively. (**e**) Hematoxylin and eosin staining of a teratoma derived from EMS-iPS cells (×20 magnification). The teratoma is composed of gut-like epithelium (endoderm), skeletal muscle (mesoderm), and melanocytes (ectoderm). (**f**) EMS-iPS cells exhibit the 46, XY, t(8;9)(p12;q33) karyotype, as determined by G-banding analysis. (**g**) PCR analysis of the expression level of the CEP110-FGFR1 fusion transcript in primary BM cells from the patient and EMS-iPS cells.

We established an iPS cell line from an EMS patient (Fig. 1A) by infecting patient-derived BM MNCs with retroviruses encoding the reprogramming factors OCT4, SOX2, KLF4, and c-MYC. The EMS-iPS cells (Fig. 1A) had the same morphology as embryonic stem cells, even after more than 20 passages, and expressed pluripotency markers (Fig. 1B and 1C, top). Semi-quantitative RT-PCR confirmed that the exogenous reprogramming genes *KLF4*, *SOX2*, *C-MYC*, and *OCT3/4* were silenced in EMS-iPS cells (Fig. 1C, bottom). Scant methylation of the *OCT3/4* and *NANOG* promoter regions was confirmed using bisulfite PCR, thus indicating successful reprogramming [25] (Fig. 1D). The capability of EMS-iPS cells to differentiate into all three germ layers was confirmed in a teratoma formation assay (Fig. 1E). At the generation of EMS-iPS cells from the patient, BM sample showed mosaic pattern of karyotype, which exhibited the additional abnormality with t(8;9)(p12;q33). EMS-iPS cells produced here showed the karyotype of t(8;9)(p12;q33), which was same karyotype with the major clone of BM cells (Fig. 1F and S1 Table). Furthermore, the same chimeric CEP110-FGFR1 fusion was confirmed using PCR of genomic DNA in patient BM and EMS-iPS cells and sequencing them (Fig. 1G and S1 Fig.). Thus, iPS cells were successfully established from the EMS patient. It is reportedly difficult to establish iPS cells from patients with hematologic malignancies [26]. Our attempts to generate more EMS-iPS cell clones using other BM samples failed.

### EMS-iPS cells exhibit augmented hematopoiesis

**Fig 2:**
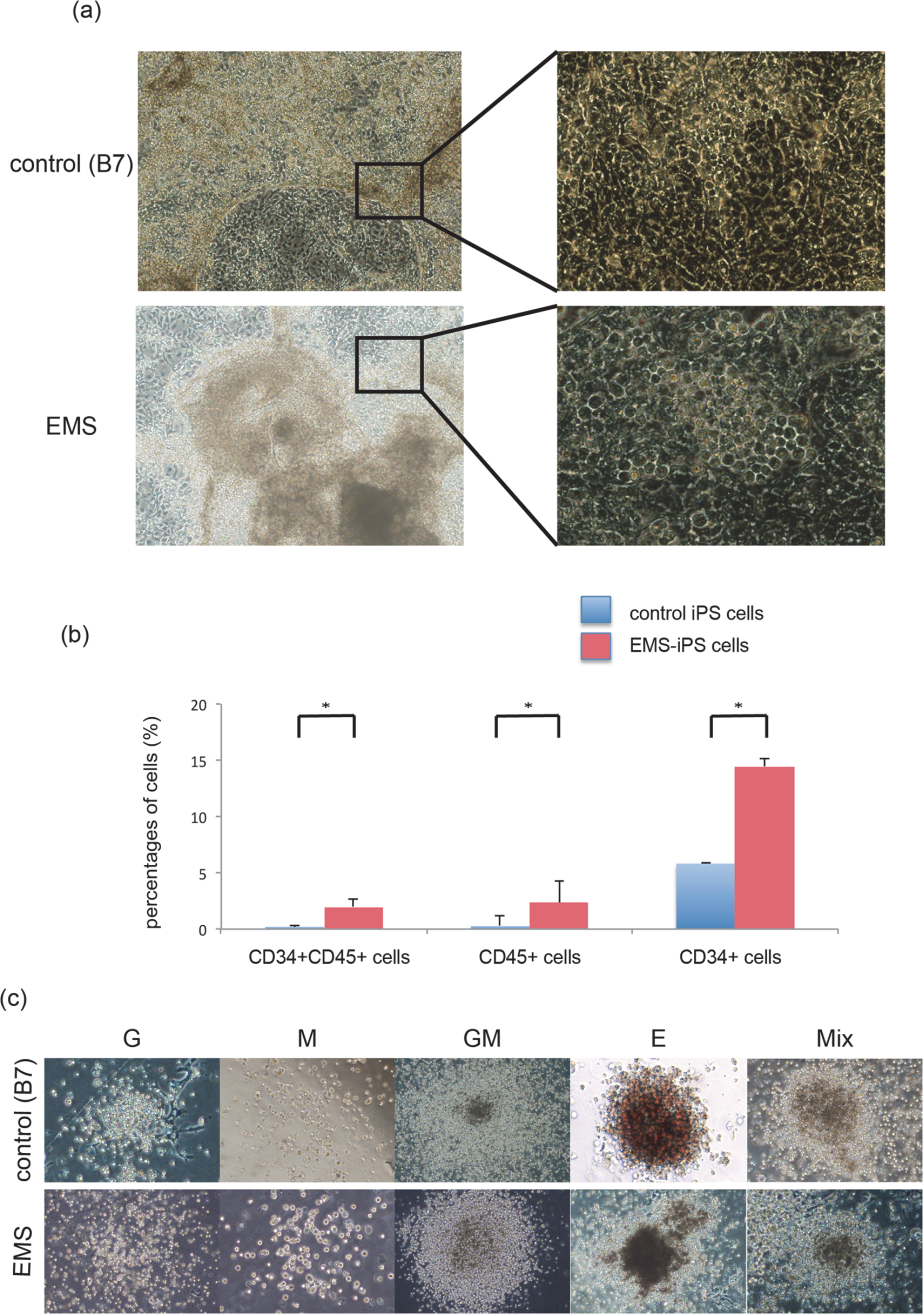
EMS-iPS cells exhibit augmented hematopoiesis. (a) Cobblestone morphology of iPS cells. Control human 201B7 iPS cells (B7) or EMS-iPS cells were co-cultured with murine AGM-S3 cells. After 12 days of co-culture, cells with a cobblestone morphology were detected at the peripheries of colonies. Images on the left are at ×100 magnification. Images on the right show higher magnification (×200 magnification) images of the boxed regions. (b) Flow cytometric analysis of cells cocultured with AGMS-3 on day 12. Cells were stained with antibodies specific CD34 and CD45. The percentages of CD34+ and /or CD45+ cells were shown (n = 3; bars represent SDs, p<0.05).

We examined the hematopoietic differentiation ability of EMS-iPS cells in comparison to that of control iPS cells, which were generated from healthy donors using common procedures. To generate hematopoietic cells from EMS-iPS cells and control iPS cells, a co-culture system with AGM-S3 cells was used, as previously reported [23] [21]. When colonies of undifferentiated EMS-iPS cells or control iPS cells were co-cultured with AGM-S3 cells, cells with a cobblestone morphology were detected at the peripheries of the colonies at day 11 or 12 (Fig. 2A). The area of these regions was similar in EMS-iPS cell colonies and control iPS cell colonies. At day 12, a similar number of cells were harvested from EMS-iPS cell colonies and control iPS cell colonies (approximately 1–5 × 10^6^ cells per 300 colonies). Then we checked CD34/CD45 expression of harvested cells. The percentage of harvest cells from EMS-iPS cells contained more CD34^+^ cells, CD34^+^CD45^+^ cells (hematopoietic progenitor) and CD45^+^ cells (blood cells) than control iPS cells as shown Fig. 2B.

Hematopoietic colonies derived from EMS-iPS cells. EMS-iPS cells and control 201B7 human iPS cells generated by expression of four factors were co-cultured with AGM-S3 cells. After 12 days, the cells derived from the iPS cells were collected. CD34+ cells were isolated for clonal hematopoietic culture. After 12 days, various types of colonies had formed. Images of G (×40 magnification), M (×40 magnification), GM (×40 magnification), E (×100 magnification), and Mix (×100 magnification) colonies are shown.

We next examined hematopoietic colony formation. CD34^+^ cells derived from EMS-iPS cells and two control iPS cell lines (201B7 and TkDA3-1) generated various hematopoietic colonies, namely, granulocyte (G), macrophage (M), granulocyte-macrophage (GM), erythroid (E), and mixed-lineage (Mix) (Table 1 and Fig. 2C). However, CD34^+^ cells derived from EMS-iPS cells produced significantly more CFUs than those derived from control iPS cells. CD34^+^ cells derived from EMS-iPS cells also generated 3.2–7.8-fold more M, E, and Mix colonies than those derived from control iPS cells (P < 0.05). The difference in the number of M colonies was particularly striking. CD34^+^ cells derived from EMS-iPS cells formed approximately 3.2–5.5-fold more M colonies than those derived from control iPS cells (Table 1). The size of each colony derived from EMS-iPS cells and control iPS cells did not significantly differ. These results demonstrate that EMS-iPS cells are more able to generate various hematopoietic progenitors, including macrophages, erythroid cells, and multipotent cells, than control iPS cells. In this respect, EMS-iPS cells are characteristic of cells in MPDs.

**Table 1:** Formation of hematopoietic colonies by CD34^+^ cells that were derived from EMS-iPS cells or control iPS cells co-cultured with AGM-S3 cells.

No. of colonies per 1 × 10^4^ CD34^+^ cells						
iPS cells	G	M	GM	E	Mix	total
EMS	17.5 ± 6.1	85.3 ± 14.5*	5.3 ± 3.9	18.5 ± 8.4*	6.3 ± 1.9*	132.8 ± 24.5*
Control 1	20.8 ± 4.9	15.5 ± 5.6	5.8 ± 0.5	3.5 ± 1.3	0.25 ± 0.5	44.8 ± 9.5
Control 2	9.3 ± 2.4	27 ± 5.6	3.3 ± 4.3	4.5 ± 1.0	0.8 ± 0.5	44.8 ± 5.0

EMS-iPS cells and control iPS cells (control 1: 201B7, control 2: TkDA3-1) were co-cultured with AGM-S3 cells. After 12 days, cells derived from iPS cells were collected. CD34^+^ cells were isolated for clonal hematopoietic culture. After 12 days, colonies were examined. The mean numbers of colonies ± SD of triplicate cultures are showed. G, granulocyte; M, macrophage; GM, granulocyte-macrophage; E, erythroid; and Mix, mixed-lineage colonies.

*p < 0.05 compared with the number of colonies formed by CD34^+^ cells derived from control iPS cells (Student’s t-test).

### TKIs suppress hematopoiesis of EMS-iPS cells

**Fig 3:**
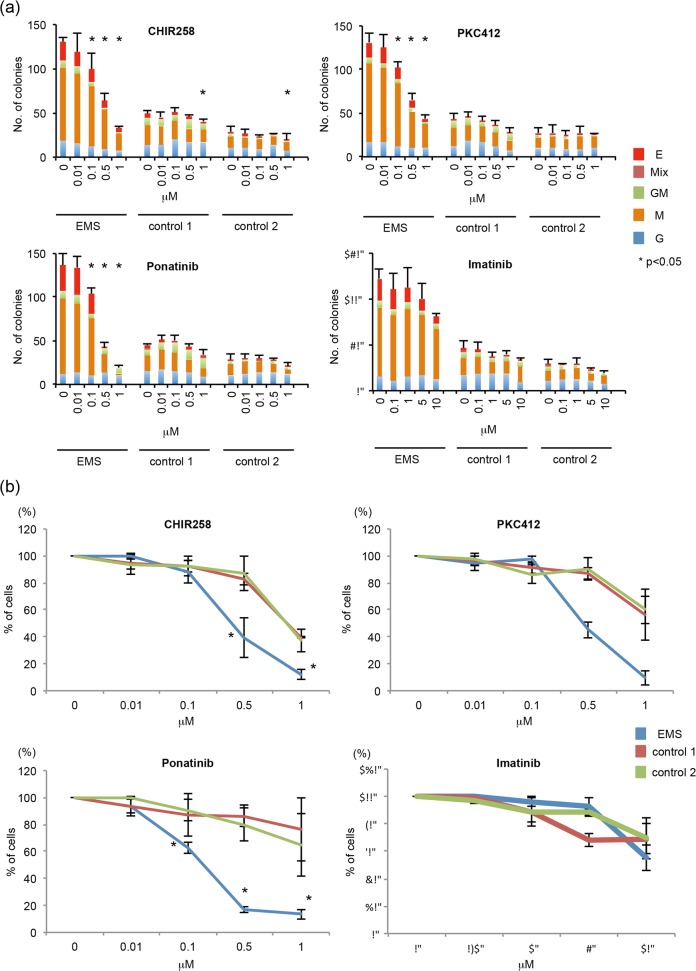
Effects of TKIs on hematopoiesis of EMS-iPS cells. (**a**) EMS-iPS cells and control iPS cells (control 1: 201B7 and control 2: TkDA3-1) generated by expression of four factors were co-cultured with AGM-S3 cells. After 12 days, cells derived from iPS cells were collected. CD34^+^ cells were isolated for clonal hematopoietic culture in the presence of various concentrations of CHIR258, PKC412, ponatinib, or imatinib. Data show mean number of colonies formed ± SD. *P < 0.05 compared to cells cultured in the absence of TKIs. (**b**) EMS-iPS cells generated by expression of four factors were co-cultured with AGM-S3 cells. After 12 days, cells derived from iPS cells were collected. CD34^+^ cells were isolated for suspension culture in the absence or presence of various concentrations of CHIR258, PKC412, ponatinib, or imatinib. Data show the mean ± SD of the percentage of viable cells compared to the number in untreated samples, which was set at 100%. *P < 0.05 compared to cells cultured in the absence of TKIs.

The TKIs CHIR258, PKC 412, and ponatinib have inhibitory effects on EMS cells *in vitro* [10] [11] [12] [13] [14]. To investigate whether these TKIs suppress the hematopoiesis of EMS-iPS cells, we examined the formation of colonies by CD34^+^ cells derived from EMS-iPS cells that were cultured with CHIR258, PKC 412, ponatinib, or imatinib. CHIR258, PKC 412, and ponatinib reduced the number of colonies (especially M colonies) formed by CD34^+^ cells derived from EMS-iPS cells in a dose-dependent manner, whereas imatinib did not (Fig. 3A). By contrast, CHIR258, PKC 412, and ponatinib did not affect the number of hematopoietic colonies formed by CD34^+^ cells derived from control iPS cells (Fig. 3A).

Next, we examined the effects of TKIs on the hematopoiesis of EMS-iPS cells in suspension cultures. CHIR258, PKC 412, and ponatinib reduced the number of CD34^+^ cells derived from EMS-iPS cells in a dose-dependent manner, and these inhibitory effects were less pronounced in control iPS cells (Fig. 3B).

Collectively, the ability of CHIR258, PKC 412, and ponatinib to preferentially suppress the differentiation of EMS-iPS cells indicates that these TKIs have a therapeutic potential in EMS.

### TKIs reduce the viability of primary PB cells isolated from the EMS patient

**Fig 4:**
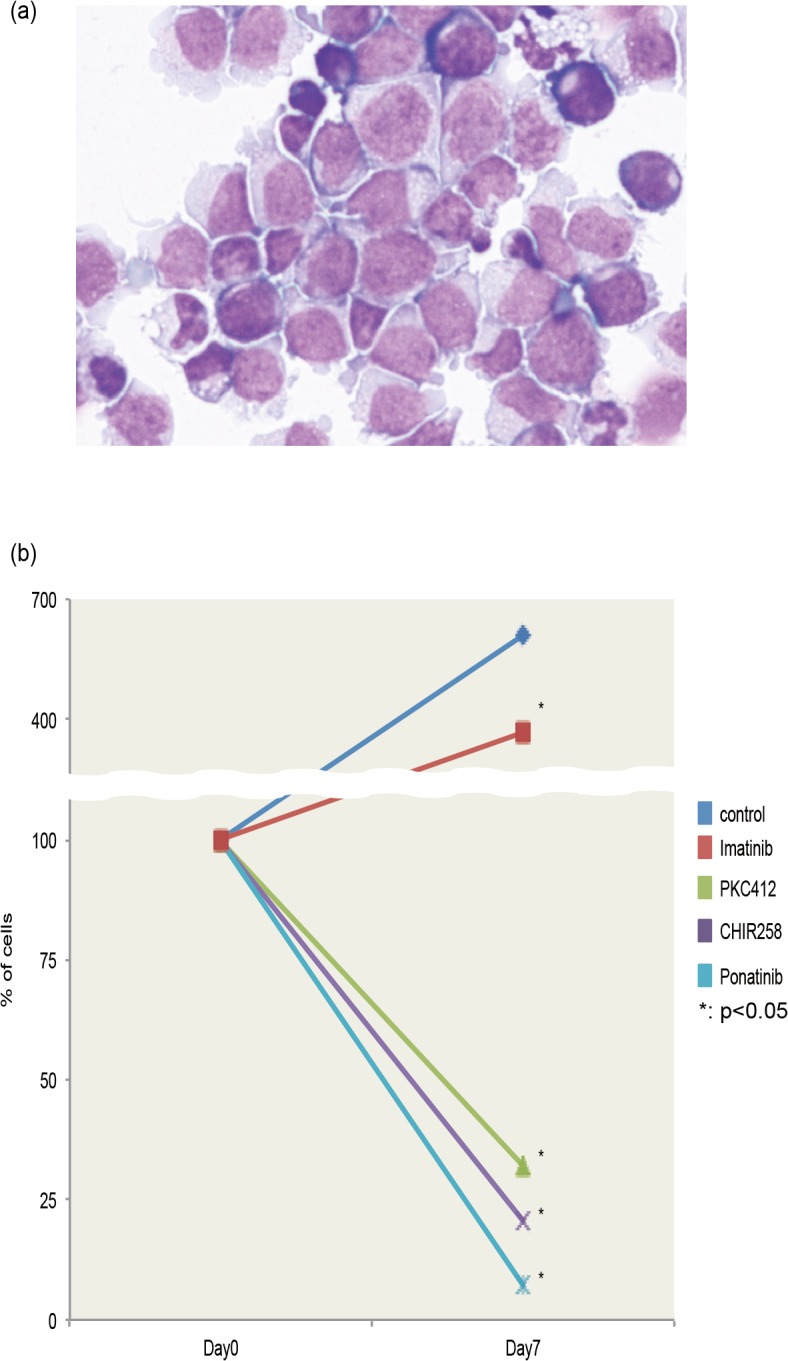
Effect of TKIs on primary PB cells of the EMS patient. (**a**) May-Grünwald Giemsa staining of a cytospin preparation of primary PB cells from the EMS patient (×100 magnification). (**b**) PM MNCs (more than 90% of which were blasts) from the EMS patient were cultured in suspension in the presence of CHIR258, PKC412, ponatinib, or imatinib for 7 days. Data show mean ± SD of the percentage of viable cells compared to the number in untreated samples, which was set at 100%. *P < 0.05 compared to cells cultured in the absence of TKIs (control).

Primary PB cells (more than 90% of which were blasts) (Fig. 4A) were isolated from the EMS patient. These cells were cultured in suspension for 7 days with 1 nM CHIR258, 1 nM PKC 412, 1 nM ponatinib, or 10 nM imatinib. The number of viable cells was determined on days 0 and 7. Over this period, the number of viable cells increased in both untreated and imatinib-treated cultures, although the number of viable cells on day 7 was significantly higher in untreated cultures than in imatinib-treated cultures. By contrast, CHIR258, PKC 412, and ponatinib significantly reduced the number of viable cells (p > 0.05) (Fig. 4B), similar to their effects on EMS-iPS cells. These results are consistent with previous reports [10] [11] [12] [13] [14] and suggest that EMS-iPS cells have similar features to those of primary PB cells isolated from the EMS patient. Therefore, EMS-iPS cells might be useful to further elucidate the mechanisms underlying EMS and to screen drugs to treat this disease.

## Discussion

In this study, we established an iPS cell clone from BM MNCs that were isolated from a patient with EMS. EMS-iPS cells had the t(8;9)(p12;q33) translocation and expressed a chimeric FGFR1/CEP110 fusion transcript, which was also detected in BM MNCs of this patient. The EMS-iPS cells fulfilled standard quality control criteria for iPS cells, including the expression of endogenous pluripotency markers, the silencing of transduced reprogramming genes, demethylation status of cells, and the ability to form teratomas. The methods we used might generate iPS cells less efficiently from BM cells isolated from EMS patients than from those isolated from healthy donors. We were only able to establish one EMS-iPS cell clone. In general, it is difficult to produce iPS cells from patients with hematologic malignancies [26], and only a few hematologic malignancy-specific iPS cell clones have been reported[27–31]. Owing to the rarity of EMS, the features of this disease are not fully understood. It is possible that the cells of the EMS patient were resistant to transduction with reprograming factors by the methods we used. Further studies are required to develop better techniques to generate hematologic malignancy-specific iPS cell clones.

EMS-iPS cells generated more hematopoietic cells including hematopoietic progenitors, especially those of a macrophage lineage, than control iPS cells. Transduction with a FGFR1 fusion gene increases the capacity of cells to differentiate into the erythroid lineage [32]. CD34^+^ cells derived from EMS-iPS cells generated colonies of various lineages, including the erythroid lineage (Table 1). These results might reflect the characters of MPD, which EMS basically had.

Although EMS-iPS cells had the same karyotype as BM cells isolated from the patient, preliminary attempts failed to immortalize EMS-iPS cells-derived hematopoietic progenitor cells. This could be explained by two possibilities. First, the culture system we used to differentiate iPS cells into hematopoietic cells might not be appropriate to observe leukemogenesis. Particular cytokines or environmental factors might be necessary that replicate the BM microenvironment of the patient. It was recently reported that NOD/SCID/IL-2Rnull mice develop AML following transplantation and engraftment of CNTRL-FGFR1-transduced CD34^+^ cells [33]. The duration from transplantation of these cells to development of MPD and AML was 7 and 15 months, respectively. EMS-iPS cells might also take a long time to give rise to AML Second possibility is that the BM sample we used to generate iPS cells had several clones, which included clones sustained in MPD status or transformed into AML. Actually, BM sample using for generation of EMS-iPS cells had a few clones (S1 Table). The karyotype of t(8;9)(p12;q33) seemed to be original clone because of the only clone existed at diagnosis. Interestingly iPS cells already generated from hematologic malignancy had the feature of MPD in their background [27–31]. It is extremely difficult to establish iPS cells from patients with *de novo* acute leukemia. Considering above reports, we might generate EMS-iPS cells from pre-leukemic MPD cells, which did not transform to AML cells although these two clones might showed same karyotype. Additional unidentified mutations or epigenetic modifications might be necessary to develop leukemic blasts from EMS-iPS cells.

EMS is rare disease with a poor prognosis and which responds poorly to chemotherapy. At present, the only cure for EMS is allogeneic stem cell transplantation. However, in the patient studied here, the disease progressed following allogeneic BM transplantation. Some drugs (TKI258, PKC412, and ponatinib) reportedly have promising effects against EMS *in vitro* [10] [11] [12] [13] [10–14], although the gene to which *FGFR1* was fused differed between these previous reports and the current study. This study showed that EMS-iPS cells could be used instead of the patient’s primary cells to study EMS because both cell types exhibited similar sensitivities to four TKIs (Fig. 4). So far it is challenging to make the model of the engraftment of hematopoietic cells from human iPS cells. But it is ideal to have data for drug sensitivity since considering the pharmacokinetics and pharmacodynamics of drug.

In general, it is difficult to obtain sufficient data about treatments for rare diseases. Our study suggests that iPS cells derived from patients with rare diseases can provide a renewable source to investigate these diseases and to develop effective treatments. Moreover, these iPS cells could be used instead of primary cells from the patient. Thus, iPS cells could be used in personalized therapies.

In conclusion, we established iPS cells from a patient with EMS. These cells exhibited characteristics of EMS. EMS-iPS cells are renewable and can therefore be used to investigate the pathophysiology of EMS and to develop an optimal therapeutic strategy. Such patient-derived iPS cells could be used in personalized therapies for many diseases, including those that are rare.

## Supporting Information

S1 Fig Schematic representation of the identified CEP-FGFR1 fusion point. The DNA sequences at the fusion border are presented in both patient BM sample and EMS-iPS cells, respectively.
(TIF)

S1 Table Karyotype of the patient and EMS-iPS cells. (DOCX)

S2 Table Primers used for PCR. (DOCX)
