# Supplementary figures and images for: Screening of Drugs to Treat 8p11 Myeloproliferative Syndrome Using Patient-Derived Induced Pluripotent Stem Cells with Fusion Gene CEP110-FGFR1
Source: PLoS One. 2015 Mar 24;10(3):e0120841. doi: 10.1371/journal.pone.0120841 (PMC4372437; doi:10.1371/journal.pone.0120841)

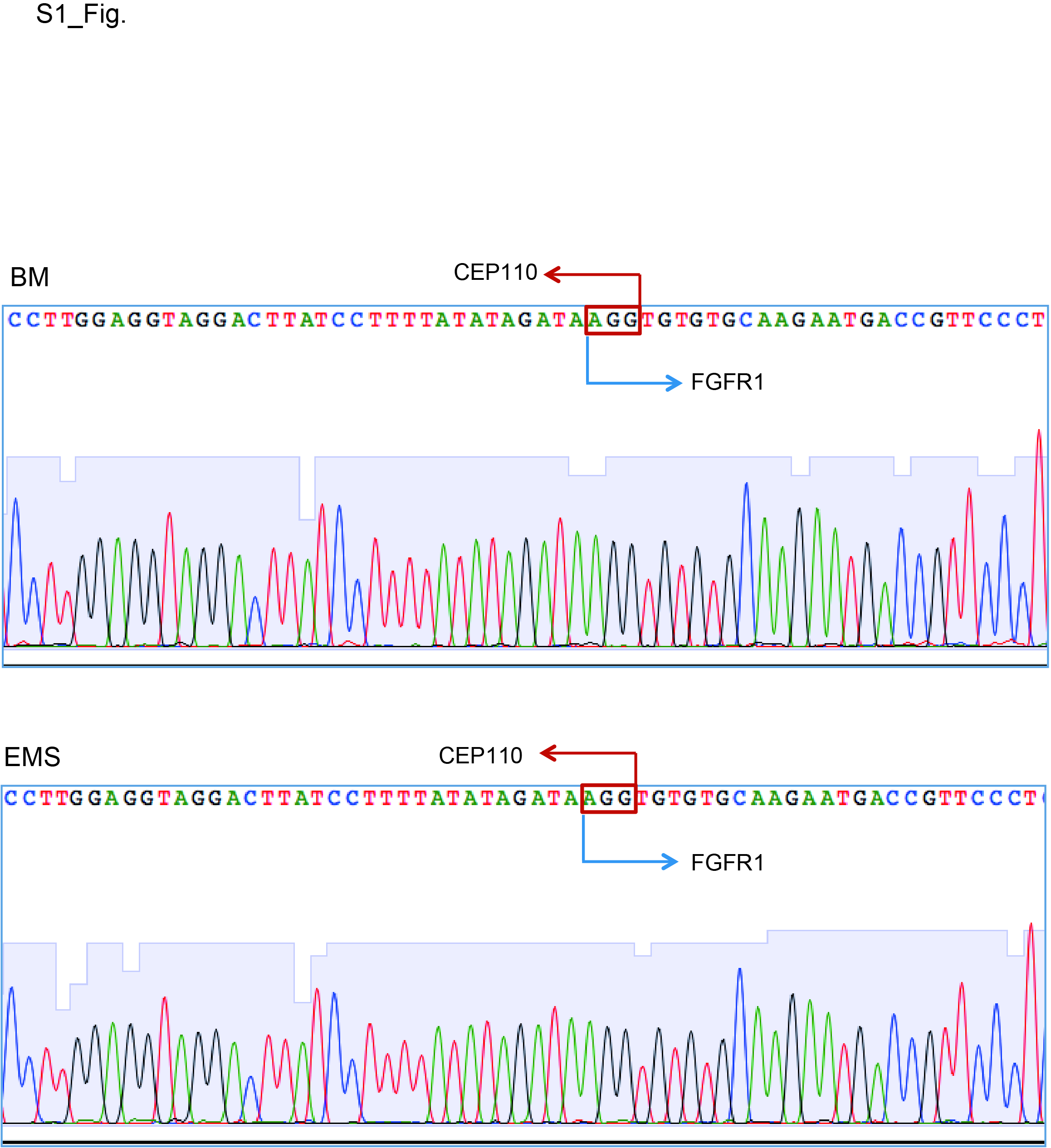

Supplement: S1 Fig — The DNA sequences at the fusion border are presented in both patient BM sample and EMS-iPS cells, respectively. (TIF) [file pone.0120841.s001.tif]
