# Supplementary material for: Screening of Drugs to Treat 8p11 Myeloproliferative Syndrome Using Patient-Derived Induced Pluripotent Stem Cells with Fusion Gene CEP110-FGFR1
Source: PLoS One. 2015 Mar 24;10(3):e0120841. doi: 10.1371/journal.pone.0120841 (PMC4372437; doi:10.1371/journal.pone.0120841)
Supplement: S1 Table — (DOCX) [file pone.0120841.s002.docx]

**S1_Table** Karyotype of the patient and EMS-iPS cells

Patient EMS-iPS cell

––––––––––––––––––––––––––––––––––––––––––––––––––––––––––––––––––––––––––––

At diagnosis 46,XY,t(8;9)(p12;q33) [20/20] -

BM sample 46, XY, t(8;9)(p12;q33) [9/20] -

for iPS cells 47, idem, +21 [4/20]

48, idem, +19, +21 [5/20]

46, idem, del(6)(q) [2/20]

- 46,XY,t(8;9)(p12;q33) [20/20]

––––––––––––––––––––––––––––––––––––––––––––––––––––––––––––––––––––––––––––
