# Supplementary material for: Screening of Drugs to Treat 8p11 Myeloproliferative Syndrome Using Patient-Derived Induced Pluripotent Stem Cells with Fusion Gene CEP110-FGFR1
Source: PLoS One. 2015 Mar 24;10(3):e0120841. doi: 10.1371/journal.pone.0120841 (PMC4372437; doi:10.1371/journal.pone.0120841)
Supplement: S2 Table — (DOCX) [file pone.0120841.s003.docx]

**S2_Table** Primers used for PCR

| reprogramming factors (semiquantitative RT-PCR) | | |
| --- | --- | --- |
| KLF4 Trans | hKLF4-S1128 | ACGATCGTGGCCCCGGAAAAGGACC |
|  | pMXs-AS3206 | TTATCGTCGACCACTGTGCTGGCG |
| KLF4 Endo | hKLF4-S1820 | AGAGTTCCCATCTCAAGGCA |
|  | hKLF4-AS2112 | TCATCGGGAAGACAGTGTGA |
| c-MYC Trans | hcMYC-F1791 | AGCAGAGGAGCAAAAGCTCATT |
|  | Viral LTR | AACCTACAGGTGGGGTCTTTCA |
| c-MYC Endo | hc-MYC_endo_F1977 | CCTCACAACCTTGGCTGAGT |
|  | hc-MYC_endo_R2222 | GGATTGAAATTCTGTGTAACTGC |
| SOX2 Trans | hSOX2-F764 | TTACCTCTTCCTCCCACTCCAG |
|  |  |  |
|  | Viral LTR | AACCTACAGGTGGGGTCTTTCA |
| SOX2 Endo | hSOX2-S1430 | GGGAAATGGGAGGGGTGCAAAAGAGG |
|  | hSOX2-AS1555 | TTGCGTGAGTGTGGATGGGATTGGTG |
| OCT4 Trans | hOCT4-F875 | ATGCACAACGAGAGGATTTTGA |
|  | Viral LTR | AACCTACAGGTGGGGTCTTTCA |
| OCT4 Endo | hOCT4-S1165 | GACAGGGGGAGGGGAGGAGCTAGG |
| CEPF2 |  | TTCCAGTGCCTATTGTGTGC |
| FGFR_ER |  | GTCTTCGGGAAGCTCATACTCAGA |
